# Variation in Large Language Model Recommendations in Challenging Inpatient Management Scenarios

**DOI:** 10.1007/s11606-025-09888-7

**Published:** 2025-10-07

**Authors:** Susan Landon, Thomas Savage, S. Ryan Greysen, Eric Bressman

**Affiliations:** 1 Department of Medicine, Perelman School of Medicine, University of Pennsylvania, Philadelphia, PA USA; 2 Leonard Davis Institute of Health Economics, University of Pennsylvania, Philadelphia, PA USA; 3 Corporal Michael J. Crescenz VA Medical Center, Philadelphia, PA USA

## Abstract

**Importance:**

Large language models (LLMs) are entering clinical workflows, yet their behavior in routine bedside decisions that lack a single “correct” recommendation remains unclear.

**Objective:**

To describe variation within and across commercially available LLMs when confronted with common, judgment-dependent inpatient medicine management scenarios.

**Design:**

Cross-sectional simulation study. Four brief vignettes requiring a binary management decision were posed to each model in five independent sessions. Six LLMs were queried: five general-purpose (GPT-4o, GPT-o1, Claude 3.7 Sonnet, Grok 3, and Gemini 2.0 Flash) and one domain-specific (OpenEvidence).

**Exposures:**

Standardized prompts describing (1) transfusion at borderline hemoglobin, (2) resumption of anticoagulation after gastrointestinal bleed, (3) discharge readiness despite a modest creatinine rise, and (4) peri-procedural bridging in a high-risk patient on apixaban.

**Main Measures:**

Primary outcomes were each model’s overall recommendation (majority across five runs) and its internal consistency (proportion of identical recommendations across runs; range 0–1). Inter-model agreement was the proportion of models giving the same recommendation.

**Results:**

A total of 120 model-vignette interactions were analyzed. Inter-model recommendations diverged in every scenario: transfuse vs observe (67% vs 33% of models), restart vs hold anticoagulation (50% vs 50%), discharge vs delay (50% vs 50%), and bridge vs no-bridge (17% vs 83%). Across five repeated queries of the same vignette, some models changed recommendations in two of five runs (internal consistency as low as 0.60). OpenEvidence was the most internally consistent and concrete in its recommendations; every other model displayed internal variability in one or more vignettes.

**Conclusions:**

For nuanced inpatient management questions, widely used LLMs produced inter- and intra-model variation in their recommendations. Clinicians should view LLM output as one perspective among many, consider sampling multiple models or re-prompting, and retain final responsibility for bedside decisions. Prospective studies are needed to test designs that surface model uncertainty and support safe integration of generative AI into complex decision-making.

**Supplementary Information:**

The online version contains supplementary material available at 10.1007/s11606-025-09888-7.

## Introduction

Large language models (LLMs) have evolved rapidly in recent years, demonstrating capabilities across multiple domains of medical practice. Early evaluations of these models, such as performance on multiple-choice United States Medical Licensing Examination (USMLE) questions,^1^ have given way to more complex testing scenarios that probe the ability of LLMs to apply clinical reasoning to open-ended case vignettes.^2,3^ While their performance in these studies has been impressive — at times exceeding that of the average physician — these simulations are not representative of the majority of real-world practice.^4^

In typical clinical settings, many decisions lack a single, discrete “correct” recommendation. Instead, they require judgment calls that balance competing risks, patient preferences, and clinical uncertainty— the art, rather than the science, of medicine. Nevertheless, many practitioners are integrating LLMs into their workflows,^5,6^ raising questions about how these models handle such ambiguity in their reasoning and decision making.

In this study, we examine how different commercially available LLMs respond to nuanced real-world management scenarios commonly encountered in inpatient medicine. By comparing their recommendations, we aim to assess variation within and across these models. Understanding these capabilities and their limitations will help clinicians, educators, and policymakers as they consider how to incorporate LLMs into inpatient care.

## Methods

### Study Design

We conducted a cross-sectional simulation study comparing the recommendations of six commercially available large language models (LLMs) when faced with four common, judgment-dependent inpatient scenarios. Each scenario required a binary management decision (e.g., transfuse vs observe). Four vignettes were drafted to represent nuanced situations in which (1) guidelines provide limited direction or available evidence does not cover the exact scenario, and (2) bedside management is known to vary among clinicians (Table 1).^7,8^ These were written in a brief format, meant to reflect the style and level of detail a typical user might present when querying an LLM.

**Table 1:** Case Vignettes

Topic	Prompt
Vignette 1: Transfusing borderline hemoglobin	“A 58 year-old male with a history of HFrEF, CAD, CKD3 admitted for acute decompensated heart failure, has been anemic the entire admission (7–7.5), and on day 6 of hospitalization his Hgb is 6.9. Should he be transfused today?”
Vignette 2: Restarting anticoagulation after bleed	“A 72 year-old female with a history of Afib (on apixaban), TIA, HTN, DM, was admitted with an upper GIB. EGD found a peptic ulcer with an actively bleeding vessel at the base, which was cauterized. On day 2 she is feeling better, there are no signs of bleeding, and she is eager to go home. Would you restart anticoagulation prior to discharge?”
Vignette 3: Discharge with lab abnormality	“A 67 year-old male with a history of colon cancer (s/p hemicolectomy with anastomosis), hypothyroidism, CKD3 (baseline creatinine 1.6–1.7), HTN/HLD, COPD, presents with fever, SOB/cough and found to have a community acquired pneumonia. After 4 days of antibiotics he is feeling much better. Labs this morning show a normal white count, and a creatinine bump (1.85 from 1.6 yesterday). He is eager to go home. Would you discharge today?”
Vignette 4: Bridging anticoagulation in high risk	“A 67 year-old female with a history of PAD, CAD (s/p CABG eight years ago; on ASA, statin), CVA (5 years ago, no residual deficit), HFpEF, DM2, Afib (on apixaban), HTN, chronic toe wound related to vascular disease, was sent in by vascular for angiogram with possible intervention. They would like to hold anticoagulation prior to the procedure. Would you bridge her peri-operatively?”

### Model Selection

We included five general-purpose LLMs — GPT-4o (OpenAI), GPT-o1 (OpenAI), Claude Sonnet 3.7 (Anthropic), Grok 3 (xAI), and Gemini 2.0 Flash (Google) — and one domain-specific model, OpenEvidence, which is trained on biomedical literature. All models were accessed via their publicly available web interfaces between March 24 and April 8, 2025. Temperature or “creativity” settings were left at each platform’s default to mirror typical clinical use.

### Prompting Protocol

Each interaction opened with the identical primer: “You are an expert hospitalist, faced with the following patient scenario. What would you do and why?” The vignette and yes/no management question immediately followed. To gauge intra-model variability, we posed each case five times in fresh sessions with cleared conversation history; cross-conversation memory features (available at the time of the study for certain OpenAI users) were inactive. No follow-up questions or clarifications were provided.

### Outcomes

The overall management recommendation (e.g., whether to transfuse) was the choice made most frequently across all five runs. A single recommendation was ascribed to each response, regardless of the strength of the recommendation (e.g. “I would definitely” and “I would lean toward” were treated the same). Internal consistency was calculated as the proportion of identical recommendations made by the model across all five runs (range 0 to 1, with 1 indicating perfect internal agreement). For each vignette, we summarize inter-model agreement by comparing overall recommendations across models. We also summarize: (1) key clinical factors from the case emphasized in the model’s reasoning, (2) additional diagnostic and management steps recommended by the model, and (3) key guidelines or trials cited, if any, by the model. The complete text of each model’s first response to every vignette is available in the Appendix.

As this study evaluated publicly accessible software without involving human participants or protected health information, it was deemed non-human-subjects research and therefore did not require Institutional Review Board review.

## Results

### Vignette 1

In a majority of repeated prompts, four of six (67%) models recommended transfusion now, while two (33%) recommended continued observation (Table 2). Median internal consistency was 0.8 (range 0.6–1.0). “Transfuse” models anchored on the restrictive 7 g/dL threshold, whereas “observe” models emphasized volume-overload risk and patient stability. The models that recommended transfusion tended to be more concrete and concise in their guidance (“transfusion is indicated.”), whereas those that recommended observation tended to be more verbose and less certain in their wording (“I might hold off.”).

**Table 2:** Responses to Vignette 1—Transfusing Borderline Hemoglobin

Model	Overall	Internal consistency	Key considerations	Management plan	Sources cited
GPT-4o	Observe	0.8	- Known cardiac disease - Chronicity - Asymptomatic - Potential for overload	- Recheck tomorrow - Monitor vitals and symptoms - Transfuse if downward trend continues	American Association of Blood Banks (AABB) guidelines
GPT-o1	Transfuse	1.0	- Hgb < 7 - Asymptomatic - Potential for overload	- Transfuse 1 unit now - Check post- transfusion CBC - Monitor volume status	TRICC, TRISS
Claude Sonnet 3.7	Observe	0.6	- Known cardiac disease - Asymptomatic - Potential for overload	- Hold off on transfusion - Investigate cause of anemia - Assess volume status - Consider cardiology consultation	None
Grok 3	Transfuse	0.6	- Hgb < 7 - Known cardiac disease, CKD - Asymptomatic - Potential for overload	- Transfuse 1 unit now, slowly; give with furosemide - Check post-transfusion CBC - Investigate cause of anemia	AABB, ACC/AHA guidelines; TRICC
Gemini 2.0	Transfuse	0.8	- Hgb < 7 - Known cardiac disease	- Transfuse now - Assess volume status - Investigate cause of anemia	None
OpenEvidence	Transfuse	1.0	- Hgb < 7 - Known cardiac disease	- Transfuse now	ACP, AABB, CHEST guidelines

### Vignette 2

Three (50%) models recommended restarting anticoagulation on discharge, and three (50%) recommended holding in a majority of prompts (Table 3). Median internal consistency was 0.8 (range 0.6–1.0). “Restart” models focused on successful endoscopic hemostasis, while “hold” models focused on healing time. Of the models that recommended holding anticoagulation, the duration ranged from 4 to 14 days, and two models (GPT-o1 and Claude Sonnet) considered bridging with low-dose prophylaxis. OpenEvidence was the lone model that did not mention the patient’s stroke risk or discuss additional management considerations (e.g. starting a proton pump inhibitor).

**Table 3:** Responses to Vignette 2—Restarting Anticoagulation After Bleed

Model	Overall	Internal consistency	Key considerations	Management plan	Sources cited
GPT-4o	Restart	0.8	- High stroke risk - Bleeding source definitively addressed - Clinically stable	- Restart full dose AC - PPI - Close outpatient follow-up - Repeat EGD later to document healing	AUGUSTUS; Danish registry data
GPT-o1	Don’t restart	0.6	- High stroke risk - High risk stigmata of bleeding on EGD - Guidelines recommend waiting at least 3 days	- Hold full-dose AC for 1 week; can consider prophylactic dose - PPI - Observe inpatient for longer, if possible	“Guidelines or expert recommendations”
Claude Sonnet 3.7	Don’t restart	1.0	- High stroke risk - Cauterized vessel needs to heal, risk of re-bleed	- Hold AC for 1–2 weeks, pending reassessment - Close outpatient follow-up - Consider low-dose aspirin - PPI	None
Grok 3	Don’t restart	0.6	- High stroke risk - Ulcer still healing, risk for rebleed	- Hold AC until 4–7 days post EGD - PPI - Follow-up within 2–3 days	ACC, ACG guidelines
Gemini 2.0	Restart	0.8	- High stroke risk - Lower risk of rebleeding given successful cautery	- Restart full-dose AC - Arrange follow-up - Consider PPI	Lay audience webpage about stroke risk
OpenEvidence	Restart	1.0	- Hemostasis achieved - Restarting anticoagulation lowers thromboembolic risk	- “Reasonable” to restart full dose AC (though could consider waiting until 7 days)	ACC guidelines; several observational studies

### Vignette 3

Across five re-prompts, three (50%) models recommended discharging today, and three (50%) recommended delaying a majority of the time (Table 4). Internal consistency was high (median 1.0, range 0.8–1.0), with only Claude and Grok wavering once. “Discharge” models framed the creatinine change as not meeting formal acute kidney injury (AKI) criteria and highlighted hospital-associated harms; “stay” models labeled the bump a possible early AKI and called for additional workup and repeat labs. Gemini consistently misapplied the AKI definition.

**Table 4:** Responses to Vignette 3—Discharge with Modest Creatinine Rise

Model	Overall	Internal consistency	Key considerations	Management plan	Sources cited
GPT-4o	Discharge	1.0	- Clinically improved - Creatinine bump mild and not unexpected - Staying in the hospital carries risk - Patient preference	- Repeat labs within 2–3 days	None
GPT-o1	Don’t discharge	1.0	- Small creatinine bump may represent early AKI	- Monitor for another day, repeat labs in 24 h - Check medication list - Assess volume status	None
Claude Sonnet 3.7	Don’t discharge	0.8	- Baseline CKD puts him at higher risk	- Monitor for another day, repeat labs in 24 h - Check med list - Assess volume status - Check additional labs (electrolytes; urinalysis to rule out UTI)	None
Grok 3	Discharge	0.8	- Does not meet criteria for AKI - Creatinine bump mild and not unexpected - Staying in the hospital carries risk - Patient preference	- Repeat labs within 2–3 days - Ensure medications are renally dosed - Ensure adequate hydration	KDIGO
Gemini 2.0	Don’t discharge	1.0	- Concerning for AKI - Risk for readmission or longer term kidney damage	- Don’t discharge - Check medication list - Assess volume status - Check additional labs (repeat Cr/BUN, UA, electrolytes, FeNa)	None
OpenEvidence	Discharge	1.0	- Clinically improved - Creatinine bump mild and not unexpected	- Ensure early follow-up with primary care	ATS, IDSA — guidelines regarding readiness for discharge in pneumonia

### Vignette 4

Over successive prompts, in a majority of cases, five (83%) models recommended holding anticoagulation without bridging, while one (17%) favored bridging (Table 5). Four models were perfectly consistent, while Grok and Gemini were less consistent (0.6). All of the models directly cited or alluded to guidelines that bridging is generally not recommended with direct oral anticoagulants (DOACs); Grok was the only model to note that this patient differed from the population studied in the BRIDGE trial. Models varied in their recommendations of timing for holding and restarting anticoagulation. Gemini uniquely warned of the risk of heparin-induced thrombocytopenia, which is a rare condition and an uncommon consideration when deciding to start low molecular weight heparin in particular.

**Table 5:** Responses to Vignette 4—Bridging Anticoagulation in High Risk

Model	Overall	Internal consistency	Key considerations	Management plan	Sources cited
GPT-4o	Don’t bridge	1.0	- High CHADS-VASc - Prior stroke 5 years ago - Bridging generally not recommended with DOACs	- Hold AC 2–3 days before procedure - Restart 1–2 days after procedure	None (though guidelines alluded to)
GPT-o1	Don’t bridge	1.0	- Prior stroke 5 years ago - Bridging generally not recommended with DOACs	- Hold AC 2–3 days before procedure - Restart ~ 1 day after procedure	None (though guidelines alluded to)
Claude Sonnet 3.7	Don’t bridge	1.0	- High CHADS-VASc - Potential bleeding complications from bridging	- Hold AC 2 days before procedure - Restart 1–2 days after procedure	None (though evidence described in general terms)
Grok 3	Bridge	0.6	- High CHADS-VASc (higher than those studied in the BRIDGE trial) - CAD and PAD raise her thrombotic risk further - Elevated HAS-BLED score	- Hold apixaban 2 days before procedure - Start LMWH 24 h after last dose; stop 24 h before procedure - Restart LMWH 1–2 days post-procedure, and then resume apixaban “once safe”	ACC, AHA, CHEST guidelines; BRIDGE trial
Gemini 2.0	Don’t bridge	0.6	- CHADS-VASc score (recommends recalculating, but does not recalculate itself) - High bleeding risk - Bridging generally not recommended with DOACs - Increased risk of heparin-induced thrombocytopenia	- Likely hold AC for 1–2 days before procedure - Patient education and shared decision making	None (but recommends looking at ACC, AHA, and ASH guidelines)
Open Evidence	Don’t bridge	1.0	- Bridging generally not recommended with DOACs	- Hold AC 1–2 days before procedure - Restart 1–2 days after procedure	ACC, AHA, American College of Chest Physicians guidelines; BRIDGE trial

## Discussion

In this simulation of four nuanced inpatient management decisions, large language models frequently provided different recommendations from one another— and showed a high degree of internal variability as well. When faced with an identical vignette five times in a row, individual models changed their recommendation up to 40% of the time, and inter-model agreement hovered around chance. In addition, there was no clear correlation between model recommendations (i.e., no two models consistently provided the same recommendations across cases).

Most published LLM studies have evaluated tasks that have an unambiguous ground truth (e.g., exam questions, guideline-concordant antibiotic choices).^9–11^ These studies are important, but overlook the fact that a significant portion of clinical practice does not have a single ground truth, whether because of uncertainty in diagnosis, management decisions not easily dictated by available evidence, or unique patient-specific factors. The present study deliberately probed that gray zone, mirroring the environment in which clinicians are already using LLMs.

Our finding of heterogeneity between LLMs in their overall recommendations for each vignette is not surprising, given that there is observed variation in provider-to-provider practice across these same scenarios. Prior studies have demonstrated variation in model accuracy in scenarios with a discrete ground truth;^11–13^ this would only be expected to amplify in cases of epistemic uncertainty. For those seeking second opinions from LLMs, as they would a colleague or consultant, these findings underscore that approaching a single model is not a shortcut to consensus medicine. Others have shown that combining models (a “collective intelligence” approach) improves diagnostic accuracy,^12^ and this may hold value in management scenarios as well.

Fewer studies have examined LLMs’ internal consistency in responses, and these generally have found a higher degree of reproducibility than we saw.^14–16^ Again, considering the nature of the scenarios we presented, this variability is not surprising, but it is illuminating. The stochastic nature of the models is such that, when asked to repeatedly reason through a complex scenario with multiple justifiable conclusions, it will not arrive at the same recommendation each time; this is a feature of probabilistic text generation. In our vignettes, that feature surfaced as clinically meaningful flip-flops (e.g., “restart” vs “do not restart” anticoagulation). Clinicians who treat an LLM as a deterministic calculator may be lulled into false certainty. It is also important for users to be aware that LLMs reflect the ambiguity of gray-zone medicine much as clinicians do; however, with brief, static inputs (as in this study) they often do not seek additional context, yielding variable recommendations. Future work should test deployments that integrate chart retrieval and require clarifying questions before issuing recommendations to determine whether reproducibility improves.

Finally, we were able to appreciate nuances of the specific models’ styles. The general-purpose models (GPT-4o, GPT-o1, Claude, Grok, Gemini) tended to narrate balanced risk–benefit analyses, to varying degrees. Grok 3’s responses were by far the longest, walking through its “thought process” in a stream-of-consciousness style; this was associated with the lowest internal consistency across all models. On the other hand, OpenEvidence — trained on biomedical literature — issued succinct, seemingly authoritative directives. For busy providers, that concreteness is attractive, but it can mask the underlying uncertainty. Interestingly, no models explicitly called out the uncertainty inherent in the vignettes presented.

Limitations include that we used vignettes which were synthetic, terse, and reflected inpatient clinical scenarios commonly encountered in the authors’ practice environment within an urban, academic health system. These prompts were intentionally designed to reflect the manner in which a busy provider might query a model in daily practice, but a richer clinical context, structured prompts (e.g., RAG pipelines that add chart data), or more objective language might reduce variability. Relatedly, the use of static, rather than iterative prompting — which is frequently used in practice and a distinctive strength of LLMs — may also affect recommendations. We did not quantify linguistic hedging or evidentiary citations, which likely influence user trust. Additionally, we only utilized five re-prompts for each LLM, which allowed us to review every model response in detail, but limited the power of the study and the ability to detect statistically significant differences in models’ internal consistency. Finally, the field evolves weekly; results obtained in April 2025 may not generalize to future model versions.

## Conclusions

Across four judgement-dependent inpatient scenarios, six widely used large language models varied in their majority recommendations and internal consistency. Clinicians using LLMs as clinical reasoning aids should treat model output as one input among many, consider re-prompting or sampling more than one model, and retain final accountability for bedside decisions. Future research should move beyond synthetic vignettes to prospective, real-world studies that (1) evaluate clinical impact, (2) surface model uncertainty transparently, and (3) identify prompt or ensemble strategies that best support frontline clinical reasoning.

## Supplementary Information

Below is the link to the electronic supplementary material.

ESM 1 (DOCX 64.1 KB)

## Data Availability

Data from this study may be made available from the corresponding author on reasonable request.
