# Supplementary material for: Variation in Large Language Model Recommendations in Challenging Inpatient Management Scenarios
Source: J Gen Intern Med. 2025 Oct 7;41(5):1286–91. doi: 10.1007/s11606-025-09888-7 (PMC13083678; doi:10.1007/s11606-025-09888-7)
Supplement: Supplementary file 1 — (DOCX 64.1 KB) [file 11606_2025_9888_MOESM1_ESM.docx]

**General prompt opening:**

**You are an expert hospitalist, faced with the following patient scenario. What would you do and why?**

**Case #1**

A 58 year old male with a history of HFrEF, CAD, CKD3 admitted for ADHF, has been anemic the entire admission (7-7.5), and on day 6 of hospitalization his Hgb is 6.9. Should he be transfused today?

**GPT-4o**

*Patient Summary:*

- *58-year-old male*
- *HFrEF (heart failure with reduced ejection fraction)*
- *CAD (coronary artery disease)*
- *CKD Stage 3*
- *Admitted for acute decompensated heart failure*
- *Chronic anemia (Hgb 7.0–7.5), now 6.9 on hospital day 6*
- *Question: Should he be transfused today?*

*Key Considerations:*

*1. Hemoglobin Threshold for Transfusion*

- *In most stable, non-bleeding medical patients, transfusion is typically considered when Hgb <7 g/dL.*
- *The AABB guidelines (American Association of Blood Banks) recommend a restrictive transfusion threshold of Hgb <7 g/dL in hospitalized, stable patients, and <8 g/dL in patients with symptomatic coronary artery disease or recent acute coronary syndrome.*

*2. Heart Failure and CAD Context*

- *In HFrEF, volume overload is a concern — transfusion could worsen pulmonary congestion.*
- *In CAD, low hemoglobin may compromise oxygen delivery to the myocardium.*
  - *If the patient is symptomatic (chest pain, dyspnea out of proportion, signs of ischemia), transfusion may be more clearly indicated even at a higher threshold.*
- *However, in chronic anemia with stable symptoms and no signs of active ischemia, we often tolerate lower hemoglobin levels.*

*3. CKD Context*

- *Anemia is common and multifactorial in CKD: reduced erythropoietin, chronic inflammation, etc.*
- *ESA (erythropoiesis-stimulating agents) may be considered for long-term management but are not helpful acutely.*
- *No indication from the scenario that the anemia is acutely worsening or due to bleeding.*

*What Would I Do?*

*I would not transfuse right away — but I would reassess closely.*

*Why:*

- *The patient is very close to the 7.0 g/dL threshold, but not clearly below.*
- *No signs of active bleeding, hemodynamic instability, or acute coronary syndrome are provided.*
- *Transfusion in a volume-overloaded patient could exacerbate heart failure, especially if not truly needed.*
- *I'd review the clinical context today:*
  - *Vital signs (HR, BP)*
  - *Oxygen requirements*
  - *Symptoms (fatigue, dyspnea, dizziness, chest pain)*
  - *Volume status (JVP, edema, weight trend)*
  - *EKG (new ischemia?)*
  - *Lactate (if worried about hypoperfusion)*
- *If truly asymptomatic and stable, I would monitor, recheck Hgb tomorrow, and ensure no ongoing losses.*

*When Would I Transfuse?*

- *Hgb drops further (e.g., <6.5 g/dL) or downward trend continues*
- *New or worsening symptoms*
- *Evidence of myocardial ischemia*
- *Preparing for a procedure*
- *Signs of hypoperfusion*

*Summary:*

*Hold off on transfusion for now, assuming patient is stable and asymptomatic. Monitor closely, reassess clinically, and avoid unnecessary volume that could worsen HF. Consider the whole picture, not just the number.*

*Would you like to draft a progress note or write up a teaching point from this case?*

Repeat attempts:

#2: don’t transfuse

#3: don’t transfuse

#4: transfuse

#5: don’t transfuse

**GPT-o1**

*In most hospitalized, hemodynamically stable patients (including those with heart failure and chronic CAD who are not actively bleeding or having active ischemia), a restrictive transfusion threshold of around 7 g/dL is appropriate based on current evidence and guidelines. Since this patient’s hemoglobin has now dropped to 6.9 g/dL, it is reasonable to transfuse 1 unit of packed red blood cells.*

*Why “restrictive” (7 g/dL) rather than “liberal”?*

- *Multiple trials (e.g., TRICC, TRISS) support that using a lower threshold (7 g/dL) in stable hospitalized patients leads to outcomes equivalent to, or better than, more liberal thresholds—particularly with respect to mortality, length of stay, and complications such as fluid overload.*
- *In a patient with heart failure, each unit of blood adds volume, increasing the risk of volume overload; hence, you want to ensure the patient truly needs the transfusion rather than transfusing at higher hemoglobin levels “just to be safe.”*

*Points to consider in this specific patient*

1. *Hemoglobin 6.9 g/dL: This is below the 7 g/dL threshold.*
2. *No active ischemia or acute bleeding: Unless he is having ongoing ischemia (chest pain, ECG changes) or overt hemorrhage, we adhere to the 7 g/dL threshold.*
3. *Monitor volume status closely: Because he has HFrEF, consider giving the packed cells slowly and possibly pairing with diuretics to avoid fluid overload.*
4. *Transfuse 1 unit at a time and reassess: Check post-transfusion hemoglobin and clinical status before deciding on additional units.*

*In short, the best evidence-based practice is to transfuse if the hemoglobin drifts below 7 g/dL in a hemodynamically stable patient who does not have active cardiac ischemia. This patient has now crossed under that threshold (6.9 g/dL), so giving at least 1 unit of packed RBCs (with close attention to volume status) is appropriate.*

Repeat attempts:

#2: transfuse

#3: transfuse

#4: transfuse

#5: transfuse

**Claude Sonnet 3.7**

*As a hospitalist considering this patient scenario, here are my thoughts on the transfusion question:*

*This 58-year-old male with multiple comorbidities (HFrEF, CAD, CKD3) admitted for acute decompensated heart failure has persistent anemia with a current hemoglobin of 6.9 g/dL on day 6 of hospitalization.*

*The transfusion decision requires careful consideration of several factors:*

*First, I'd assess for symptoms of anemia - fatigue, dyspnea, chest pain, tachycardia, or hypotension that might be attributed to the low hemoglobin rather than his heart failure.*

*For a patient with underlying cardiovascular disease like HFrEF and CAD, I would generally consider transfusion at a higher hemoglobin threshold (around 8 g/dL) than for patients without such conditions, as these patients may tolerate anemia poorly and could experience worsening cardiac ischemia.*

*However, transfusion in heart failure carries risks - volume overload could exacerbate the ADHF that prompted admission. This patient is still being treated for heart failure, making this particularly relevant.*

*Before transfusing, I would:*

1. *Investigate the cause of anemia if not already known*
2. *Evaluate for active bleeding*
3. *Consider iron studies to determine if iron deficiency is present*
4. *Assess the patient's volume status and current heart failure treatment*

*If the patient is symptomatic from anemia and hemodynamically stable, a cautious approach would be to transfuse 1 unit of packed red blood cells with close monitoring and potentially pre-treatment with IV diuretics to prevent volume overload.*

*If the patient is asymptomatic and his heart failure is improving, I might hold off on transfusion and continue to monitor, while addressing any underlying causes of anemia.*

*I'd also consider consultation with cardiology for complex cases like this where the risks and benefits must be carefully weighed.*

Repeat attempts:

#2: don’t transfuse

#3: transfuse

#4: transfuse

#5: don’t transfuse

**Grok 3***As a hospitalist evaluating this 58-year-old male patient with heart failure with reduced ejection fraction (HFrEF), coronary artery disease (CAD), chronic kidney disease stage 3 (CKD3), and acute decompensated heart failure (ADHF), the decision to transfuse for a hemoglobin (Hgb) of 6.9 g/dL on day 6 of admission requires a careful assessment of risks, benefits, and clinical context. Let’s break this down step-by-step:*

*### Key Clinical Considerations*

*1. **Severity of Anemia**:*

*- Hgb of 6.9 g/dL is significantly below the typical threshold for transfusion in most guidelines (e.g., 7 g/dL in restrictive strategies), though this is not an absolute rule.*

*- The patient has been persistently anemic (7-7.5 g/dL) throughout the admission, with a drop to 6.9 g/dL, suggesting either ongoing blood loss, hemolysis, or inadequate production (possibly exacerbated by CKD3 and inflammation from ADHF).*

*2. **Symptoms and Hemodynamic Status**:*

*- In ADHF, anemia can worsen tissue oxygenation, increase cardiac workload, and impair recovery. If the patient is symptomatic (e.g., dyspnea at rest, fatigue, tachycardia, hypotension, or signs of poor perfusion like altered mental status or lactic acidosis), transfusion may be more urgent.*

*- Without symptoms, the decision hinges on whether the anemia is contributing to ongoing heart failure decompensation despite diuresis and other therapies.*

*3. **Comorbidities**:*

*- **HFrEF**: Patients with systolic dysfunction have reduced cardiac reserve. Severe anemia could exacerbate ischemia or strain, especially with a history of CAD.*

*- **CAD**: Anemia reduces oxygen delivery, potentially risking myocardial ischemia, particularly if there’s active chest pain or ECG changes.*

*- **CKD3**: This limits erythropoietin production, contributing to chronic anemia, and may increase transfusion requirements over time.*

*4. **ADHF Context**:*

*- By day 6, the patient should ideally be stabilizing with diuresis and medical optimization (e.g., ACE inhibitors, beta-blockers, etc., as tolerated). Persistent anemia could hinder this progress, but transfusion carries risks in heart failure patients, including volume overload.*

*5. **Cause of Anemia**:*

*- The etiology isn’t specified. Potential causes include gastrointestinal bleeding (common in HF patients on antiplatelets/anticoagulants), hemodilution from fluid overload, or bone marrow suppression (e.g., from CKD or malnutrition). This impacts the decision—if there’s active bleeding, transfusion is more likely indicated alongside addressing the source.*

*### Evidence-Based Guidance*

*- **Restrictive Transfusion Strategy**: Guidelines (e.g., AABB, ACC/AHA) recommend a Hgb threshold of 7 g/dL for most hospitalized patients, including those with stable cardiovascular disease, unless symptomatic or hemodynamically unstable. For patients with acute coronary syndromes, a threshold of 8 g/dL is often considered.*

*- **Heart Failure Nuance**: Studies like the TRICC trial and subsequent analyses suggest restrictive strategies (Hgb < 7 g/dL) are safe in critically ill patients, but HF patients were underrepresented. Observational data in HF suggest anemia worsens outcomes, but transfusion doesn’t always improve mortality and may increase pulmonary edema risk.*

*### My Recommendation*

*I would **consider transfusing 1 unit of packed red blood cells (PRBCs) today**, but only after further evaluation, with the following approach:*

*1. **Assess Symptoms and Stability**:*

*- Check vital signs (e.g., heart rate, blood pressure, oxygen saturation) and symptoms. If the patient has tachycardia (>100 bpm), hypoxia (<90% on room air), or signs of ischemia (chest pain, new ECG changes), transfusion is warranted to improve oxygen delivery.*

*- If asymptomatic and hemodynamically stable, I’d lean toward holding off unless the anemia is clearly impeding ADHF recovery.*

*2. **Evaluate Volume Status**:*

*- In ADHF, transfusion can precipitate fluid overload. Ensure the patient is euvolemic (e.g., JVP, lung exam, daily weights, BNP trending down) after diuresis. If still volume-overloaded, I’d avoid transfusion unless critical, as it could worsen pulmonary edema.*

*3. **Investigate Anemia Cause**:*

*- Order a workup if not already done: reticulocyte count, iron studies, ferritin, B12/folate, lactate dehydrogenase, haptoglobin, and stool guaiac. If there’s evidence of bleeding (e.g., melena, dropping Hgb trend), transfusion may be needed alongside endoscopy or other interventions.*

*4. **Transfusion Logistics**:*

*- If proceeding, transfuse 1 unit PRBCs slowly (over 2-4 hours) with close monitoring for volume overload (e.g., respiratory status, urine output). Co-administer IV furosemide (e.g., 20-40 mg) pre- or post-transfusion if there’s concern for fluid retention, though evidence for this is mixed.*

*- Recheck Hgb post-transfusion (target ~7.5-8 g/dL, not higher, to avoid overcorrection).*

*### Why This Approach?*

*- **Benefit**: A Hgb of 6.9 g/dL risks inadequate oxygen delivery, especially with HFrEF and CAD, potentially prolonging ADHF recovery. Transfusion could improve symptoms and tissue perfusion.*

*- **Risk Mitigation**: A single unit, given cautiously, balances this benefit against the risk of volume overload or transfusion reactions. The restrictive threshold (7 g/dL) is a guide, not a mandate—clinical judgment trumps strict cutoffs here.*

*- **Contextual Nuance**: Day 6 suggests the acute phase of ADHF should be resolving. If anemia is now the bottleneck, addressing it makes sense; if not, it can wait.*

*### Final Call*

*Without more data (symptoms, vitals, volume status), I’d err toward transfusion if there’s any sign of instability or ischemia, but hold off if the patient is stable and improving. I’d also expedite the anemia workup to guide long-term management. What’s the patient’s current clinical status—any symptoms or recent trends you can share? That’d refine this further.*

Repeat attempts:

#2: transfuse

#3: don’t transfuse

#4: transfuse

#5: transfuse

**OpenEvidence**

*Yes, the patient should be transfused today.*

*The American College of Physicians (ACP) recommends a restrictive red blood cell transfusion strategy with a hemoglobin threshold of 7 to 8 g/dL in hospitalized patients with coronary heart disease.^[1]^ This patient, with a hemoglobin level of 6.9 g/dL, falls below this threshold, indicating the need for transfusion.*

*Additionally, the American Association of Blood Banks (AABB) guidelines suggest using a restrictive transfusion threshold of 7 g/dL in most patients, including those in ICUs, and 8 g/dL in patients with preexisting cardiovascular disease.^[2]^ Given the patient's history of coronary artery disease and heart failure, a hemoglobin level of 6.9 g/dL warrants transfusion.*

*Furthermore, the American College of Chest Physicians (CHEST) suggests against a restrictive RBC transfusion strategy in critically ill patients with acute coronary syndrome, indicating that a more liberal approach may be beneficial in such cases.^[3]^*

*In summary, considering the patient's hemoglobin level, underlying cardiovascular disease, and the guidelines from ACP, AABB, and CHEST, transfusion is indicated to improve oxygen delivery and reduce the risk of adverse cardiovascular events.*

Repeat attempts:

#2: transfuse

#3: transfuse

#4: transfuse

#5: transfuse

**Gemini (2.0 Flash)**

***Patient Summary:***

- ***Patient:*** *58-year-old male*
- ***PMH:*** *HFrEF (Heart Failure with Reduced Ejection Fraction), CAD (Coronary Artery Disease), CKD3 (Chronic Kidney Disease Stage 3)*
- ***Presenting Complaint:*** *ADHF (Acute Decompensated Heart Failure)*
- ***Current Issue:*** *Anemia (Hgb 6.9 g/dL) on day 6 of hospitalization, baseline Hgb 7-7.5.*

***Analysis:***

1. ***Context:***
   - *This patient has multiple comorbidities, including significant cardiac and renal issues.*
   - *Anemia is common in CKD and HFrEF, and it can worsen heart failure symptoms.*
   - *The patient has been consistently anemic during this hospitalization, with a concerning drop in hemoglobin on day 6.*
2. ***Decision-Making:***
   - *In patients with cardiac disease, especially HFrEF and CAD, maintaining adequate oxygen-carrying capacity is crucial. Anemia can exacerbate myocardial ischemia and worsen heart failure.*
   - *The hemoglobin level of 6.9 g/dL is generally considered a transfusion trigger, especially in patients with cardiovascular disease.*
   - *Given the patient's HFrEF and CAD, the risk of worsening cardiac symptoms from anemia outweighs the risks of transfusion.*
   - *It is very important to consider the patients current clinical state, is the patient symptomatic from the anemia? Symptoms of anemia include fatigue, shortness of breath, and chest pain. If the patient is symptomatic the need for transfusion is increased.*
3. ***Action:***
   - *Yes, a blood transfusion is warranted.*
   - *It is crucial to monitor the patient closely for signs of transfusion-related complications, such as volume overload, especially given the patient's HFrEF.*
   - *Further investigation into the cause of the Anemia is warranted. Common causes in this patient population include CKD associated anemia, and anemia of chronic disease.*
   - *evaluation of iron stores could be useful.*

***Rationale:***

- *Transfusing this patient will improve oxygen delivery to the myocardium, reducing the risk of ischemia and improving cardiac function.*
- *The patient's HFrEF and CAD make him more susceptible to complications from anemia.*
- *By addressing the anemia, we hope to reduce symptoms, and improve the patients clinical state.*

***In summary:***

*This patient's hemoglobin level and cardiac history indicate a need for a red blood cell transfusion. Careful monitoring for complications is essential.*

Repeat attempts:

#2: transfuse

#3: don’t transfuse

#4: transfuse

#5: transfuse

**Case #2**

A 72 year old female with a hx of Afib (on apixaban), TIA, HTN, DM, was admitted with an upper GIB. EGD found a peptic ulcer with an actively bleeding vessel at the base, which was cauterized. On day 2 she is feeling better, there are no signs of bleeding, and she is eager to go home. Would you restart anticoagulation prior to discharge?

**GPT-4**

*In this case, I would restart anticoagulation prior to discharge, with careful consideration of timing, patient risk factors, and close follow-up.*

*Here's why:*

*🧠 Clinical Reasoning*

*1. Indication for anticoagulation remains strong*

- *The patient has atrial fibrillation with prior TIA, which puts her at very high thromboembolic risk (CHA₂DS₂-VASc score ≥5).*
- *Without anticoagulation, her annual stroke risk is likely >6-10%, and possibly even higher in the short term due to temporary hypercoagulability after acute illness.*

*2. Bleeding source has been definitively treated*

- *She had an upper GI bleed from a peptic ulcer with an actively bleeding vessel, but it was successfully cauterized on EGD.*
- *She is now stable, hemodynamically well, no signs of recurrent bleeding.*

*⏱️ When to Restart Apixaban?*

- *Guidelines and studies (e.g., Danish registry data, AUGUSTUS, and expert consensus) support restarting DOACs within 3-7 days after endoscopically treated upper GI bleeds.*
- *In this case, by hospital day 2, she is stable and without bleeding—so restarting either on day 2 or 3 is appropriate, assuming no other contraindications.*

*🩺 Practical Plan*

1. *Restart apixaban prior to discharge (e.g., later on hospital day 2 or early day 3), especially if:*
   - *There are no signs of ongoing bleeding*
   - *Hemoglobin is stable*
   - *The ulcer was treated definitively*
   - *She can tolerate oral medications*
2. *Start with full dose, unless there’s a reason to dose-reduce (e.g., CrCl <30, low weight, drug interactions).*
3. *Coordinate close outpatient follow-up, ideally with:*
   - *PCP or cardiology in 1 week*
   - *Repeat GI follow-up to ensure ulcer healing if needed*
4. *Gastroprotection: Start/continue a PPI, ideally long-term if ulcer was NSAID- or idiopathic-related.*

*🛑 What if you don’t restart?*

- *You may be preventing rebleeding in the short term, but putting her at high risk of cardioembolic stroke, especially with her TIA history.*

*📚 Summary*

*Yes, I would restart apixaban prior to discharge, likely on day 2 or 3, given:*

- *High thromboembolic risk*
- *Definitively treated bleeding source*
- *Clinical stability*

*The benefits of resuming anticoagulation likely outweigh the short-term risk of rebleeding in this scenario.*

*Let me know if you'd like a one-liner or discharge summary too.*

Repeat attempts:

#2: restart

#3: restart

#4: restart

#5: don’t restart

**GPT-o1**

*When deciding when to resume anticoagulation after a high‐risk GI bleed, the goal is to strike a balance between preventing thromboembolism and avoiding re-bleeding. In an older patient with atrial fibrillation (especially with a history of TIA), it is risky to leave her off anticoagulation for too long; however, resuming too early raises the risk of repeat GI hemorrhage. Key points include:*

1. *Risk of Re-Bleeding vs. Stroke*
   - *She has a high stroke risk (AF plus prior TIA), so prolonged interruption of anticoagulation increases her chance of an ischemic event.*
   - *She also had high‐risk stigmata of bleeding (an actively bleeding vessel requiring cautery). Such lesions have a greater chance of early re-bleeding if anticoagulation is restarted immediately.*
2. *Typical Timing to Resume Anticoagulation*
   - *For patients with high‐risk endoscopic stigmata (e.g., an active bleed, visible vessel, or large ulcer), many experts and guidelines suggest waiting at least 3 days—and often up to 5–7 days—before resuming full anticoagulation.*
   - *Since she is already at Day 2 without signs of recurrent bleeding, you are just past the early re-bleeding window but still on the lower end of that usual 3–5 day range.*
3. *Practical Approach*
   - *Obtain GI input on re-bleeding risk. Confirm that the ulcer appears controlled (often assessed by endoscopic findings, hemoglobin stability, and clinical course).*
   - *Consider a brief in-hospital observation (e.g., through Day 3 or 4) before restarting, especially in someone who just had a high‐risk lesion treated.*
   - *If she remains stable, resume her anticoagulant (apixaban) prior to discharge—rather than leaving her unprotected in the outpatient setting. Starting it in-house also allows you to watch for any immediate re-bleeding.*
4. *Why Not Delay Too Long?*
   - *Patients with AF plus prior TIA or stroke have a significant risk of thromboembolic events if anticoagulation is held for a prolonged period. Delaying beyond a week can substantially increase stroke risk.*
5. *Concurrent Strategies*
   - *Ensure she is on a proton pump inhibitor (PPI) to reduce the risk of recurrent peptic ulcer bleeding.*
   - *Provide close outpatient follow-up with both cardiology and gastroenterology after discharge.*

*Bottom Line: In a high‐risk patient like this—especially with prior TIA—most would restart apixaban fairly soon (around 3–5 days post‐bleed) once bleeding is controlled. On Day 2, you are somewhat early, but she is eager to go home. If she truly remains stable by Day 3 (no drop in hemoglobin, no re-bleeding signs) and GI agrees, restarting anticoagulation before discharge (so you can observe for 24 hours in-house) is reasonable. This avoids unnecessarily prolonged exposure to stroke risk and ensures safe re-initiation under direct supervision.*

Repeat attempts:

#2: don’t restart

#3: restart

#4: don’t restart (says wait til day 7)

#5: don’t restart

**Claude 3.7 Sonnet**

*Thank you for this interesting case. As a hospitalist, I need to carefully consider the competing risks of thromboembolism versus recurrent bleeding in this patient.*

*Key considerations for this 72-year-old female with upper GI bleed:*

1. *High thromboembolic risk given her history of:*
   - *Atrial fibrillation*
   - *Previous TIA*
   - *Multiple comorbidities (HTN, DM) that increase stroke risk*
2. *High bleeding risk factors:*
   - *Recent active upper GI bleed with visible vessel*
   - *Recently cauterized ulcer (day 2 post-procedure)*
   - *Age 72*

*In this case, I would not restart apixaban prior to discharge. Here's my reasoning:*

*The patient has only been stable for one day after an actively bleeding ulcer that required intervention. While her stroke risk is significant, the immediate risk of rebleeding likely outweighs the short-term risk of thromboembolism. The cauterized vessel is still healing, and anticoagulation at this early stage could lead to rebleeding.*

*My management plan would include:*

1. *Discharge without anticoagulation initially*
2. *Ensure aggressive PPI therapy (IV PPI prior to discharge, then high-dose oral PPI)*
3. *Schedule close follow-up in 1-2 weeks*
4. *Plan for anticoagulation resumption after approximately 7-14 days, depending on reassessment*
5. *Consider a bridge with low-dose aspirin if the patient and consulting cardiologist are extremely concerned about stroke risk*
6. *Explain the rationale clearly to the patient, emphasizing this is temporary*

*This approach balances the need to prevent dangerous rebleeding in the short term while planning for appropriate stroke prevention once the ulcer has had more time to heal.*

Repeat attempts:

#2: don’t restart

#3: don’t restart

#4: don’t restart

#5: don’t restart

**Grok 3**

*As an expert hospitalist, I’ll walk through this scenario systematically to determine whether to restart anticoagulation prior to discharge.*

*This 72-year-old female has a history of atrial fibrillation (Afib) on apixaban, transient ischemic attack (TIA), hypertension (HTN), and diabetes mellitus (DM). She was admitted for an upper gastrointestinal bleed (GIB), and an esophagogastroduodenoscopy (EGD) identified a peptic ulcer with an actively bleeding vessel, which was successfully cauterized. By hospital day 2, she’s feeling better, with no signs of rebleeding, and is eager to go home. The key question is whether to restart apixaban before discharge.*

*First, let’s assess her bleeding risk post-intervention. The peptic ulcer was actively bleeding, but cauterization is an effective endoscopic hemostatic measure. Studies, such as those reflected in gastroenterology guidelines (e.g., American College of Gastroenterology), show that successful endoscopic hemostasis for a bleeding ulcer, especially without high-risk stigmata like a visible vessel or adherent clot on re-evaluation, significantly reduces the rebleeding risk. Since there are no signs of bleeding on day 2, this suggests the intervention was successful, and her immediate bleeding risk is low. However, restarting anticoagulation could theoretically increase the chance of rebleeding, so timing and context matter.*

*Next, consider her thrombotic risk. She has Afib with a history of TIA, which places her at elevated risk for thromboembolism. Using the CHA₂DS₂-VASc score: age 72 (+2), female (+1), HTN (+1), DM (+1), and prior TIA (+2) give her a score of 7. This correlates with an annual stroke risk of approximately 7-9% without anticoagulation, per cardiology literature (e.g., American Heart Association guidelines). Apixaban, a direct oral anticoagulant (DOAC), was prescribed to mitigate this risk, and stopping it long-term isn’t an option given her history.*

*Balancing these risks requires a nuanced approach. Guidelines from the American College of Cardiology and gastroenterology societies suggest that for patients with a high thrombotic risk (CHA₂DS₂-VASc ≥ 2) and a resolved GIB, anticoagulation should typically resume once hemostasis is achieved, often within 1-7 days, depending on bleeding severity and endoscopic findings. Here, the bleed was controlled on day 1, and by day 2, she’s stable. The short duration off anticoagulation (1-2 days) likely hasn’t significantly increased her stroke risk yet, but prolonged cessation would.*

*Given her stability and the successful cauterization, I’d restart apixaban prior to discharge on day 2. The rationale is twofold: (1) her bleeding risk is minimized with no ongoing hemorrhage and effective endoscopic therapy, and (2) her high thrombotic risk necessitates prompt resumption to prevent a stroke or TIA recurrence. However, I’d take precautions: ensure her hemoglobin is stable (e.g., no significant drop from baseline), confirm no melena or hematemesis, and prescribe a proton pump inhibitor (PPI) like pantoprazole 40 mg daily to reduce ulcer rebleeding risk, which DOACs can exacerbate.*

*Before discharge, I’d counsel her on signs of rebleeding (e.g., black stools, vomiting blood) and ensure close follow-up with her primary care physician or cardiologist within a week. If there were any doubts about hemostasis—say, a large ulcer or incomplete cauterization—I’d delay apixaban for another day or two and monitor longer, but that’s not the case here.*

*In summary, I’d restart apixaban prior to discharge on day 2 because her bleeding risk is low post-cauterization, and her thrombotic risk is substantial. This balances the need to prevent stroke with minimizing GIB recurrence, supported by her clinical stability and adjunctive PPI therapy.*

Repeat attempts:

#2: don’t restart

#3: don’t restart

#4: restart

#5: don’t restart

**OpenEvidence**

*In the case of a 72-year-old female with atrial fibrillation (AF) on apixaban, who was admitted with an upper gastrointestinal bleed (UGIB) and had a peptic ulcer cauterized, the decision to restart anticoagulation prior to discharge should be carefully considered.*

*The American College of Cardiology (ACC) recommends that for most cases of gastrointestinal bleeding, it is reasonable to reinitiate oral anticoagulation (OAC) once hemostasis has been achieved.^[1]^ This is supported by evidence indicating that resuming anticoagulation after a GI bleed is associated with a lower risk of thromboembolism and mortality, despite an increased risk of recurrent bleeding.^[1-4]^*

*The optimal timing for resumption of anticoagulation is not definitively established, but several studies suggest that resuming anticoagulation between 7 and 14 days after the bleeding event may balance the risks of recurrent bleeding and thromboembolism.^[2-3][5]^*

*Given that the patient is stable and eager to go home, and assuming hemostasis has been confirmed, it would be reasonable to consider restarting anticoagulation. However, it may be prudent to delay resumption until approximately 7 days post-bleed to minimize the risk of recurrent bleeding, while still protecting against thromboembolic events.^[2-3][5]^*

*In summary, restarting anticoagulation prior to discharge is reasonable, but it may be optimal to wait until around 7 days post-bleed to balance the risks of recurrent bleeding and thromboembolism. This decision should be individualized based on the patient's thromboembolic risk, bleeding risk, and overall clinical stability.*

Repeat attempts:

#2: restart

#3: restart

#4: restart

#5: restart

**Gemini**

***Patient Summary:***

- ***Patient:*** *72-year-old female*
- ***PMH:*** *Atrial fibrillation (on apixaban), TIA, hypertension, diabetes mellitus*
- ***Presenting Complaint:*** *Upper gastrointestinal bleed (GIB)*
- ***Intervention:*** *Endoscopic cauterization of a bleeding peptic ulcer*
- ***Current Status:*** *Day 2 post-procedure, no signs of bleeding, feeling better, eager for discharge.*

***Analysis:***

1. ***Balancing Risks:***
   - *The patient has a significant risk of thromboembolic events due to her atrial fibrillation and prior TIA. Apixaban is crucial for stroke prevention.*
   - *However, she also has a recent history of a major GI bleed, and restarting anticoagulation too soon carries a risk of rebleeding.*
2. ***Timing of Anticoagulation Restart:***
   - *Guidelines generally recommend restarting anticoagulation after a GI bleed once hemostasis is achieved and the risk of rebleeding is low.*
   - *The optimal timing is a balance between preventing thromboembolism and minimizing the risk of rebleeding.*
   - *Since the bleeding vessel was cauterized, and there are no signs of rebleeding, the risk of recurrent bleeding is reduced.*
3. ***Factors to Consider:***
   - *The severity of the initial bleed.*
   - *The effectiveness of the endoscopic intervention.*
   - *The patient's individual risk factors for thromboembolism.*
   - *The patient's kidney function, as apixaban is renally cleared.*
   - *The patients tolerance of oral medications.*

***Action:***

- ***Short-term:*** *It is likely safe to restart the Apixaban in this patient prior to discharge.*
- *It is very important to have a discussion with the patient regarding the risk of re-bleeding, and the risk of stroke.*
- *Ensure that the patient is tolerating oral medications.*
- *Follow up with the patient should be arranged to monitor for any signs of re-bleeding.*
- *Consider a proton pump inhibitor for continued ulcer prevention.*

***Rationale:***

- *The patient's risk of stroke from untreated atrial fibrillation is significant. ^1^* [*1. What you may not know about AFib and stroke - UChicago Medicine     www.uchicagomedicine.org*](https://www.uchicagomedicine.org/forefront/heart-and-vascular-articles/what-you-may-not-know-about-afib-and-stroke)
- *The endoscopic cauterization appears to have been successful, and the risk of rebleeding is likely decreased.*
- *Restarting anticoagulation prior to discharge will provide the patient with the most optimal stroke prevention.*
- *The risk of re-bleeding is decreased after 48 hours post endoscopic hemostasis.*

***In summary:***

*Restarting apixaban prior to discharge is likely appropriate in this case, provided there are no signs of rebleeding and the patient understands the risks and benefits. Close follow-up is essential.*

Repeat attempts:

#2: restart

#3: don’t restart

#4: restart

#5: restart

**Case #3**

A 67 year old male with a history of colon cancer (s/p hemicolectomy with anastomosis), hypothyroidism, CKD3 (baseline creatinine 1.6-1.7), HTN/HLD, COPD, presents with fever, SOB/cough and found to have a community acquired pneumonia. After 4 days of abx he is feeling much better. Labs this morning show a normal white count, and a creatinine bump (1.85 from 1.6 yesterday). He is eager to go home. Would you discharge today?

**GPT-4**

*This is a great real-world scenario that reflects the kind of nuanced decision-making hospitalists face every day. Here's how I'd approach it:*

*Summary of Key Factors*

- *67M with multiple comorbidities: colon cancer (post-surgical), CKD3, COPD, HTN, HLD, hypothyroidism.*
- *Admitted with CAP — now clinically improved after 4 days of antibiotics.*
- *Subjective status: Feels much better, eager for discharge.*
- *Lab trend: WBC normalized. Creatinine increased from 1.6 to 1.85 mg/dL (known CKD3).*

*Decision Framework*

*I'd consider three domains before deciding on discharge:*

*1. Clinical Stability*

- *Symptoms improving: Check — he feels better.*
- *Vital signs stable? We need to ensure no fevers for 24-48 hours, stable BP, HR, RR, O2 sats (especially with COPD), and no tachypnea/hypoxia. If he's weaned to room air or on home baseline O2, that would support readiness.*
- *Able to ambulate, eat, and take oral meds? If yes — another green light.*

*2. Renal Function*

- *Creatinine bump from 1.6 to 1.85 in CKD3 — that’s a small relative increase (~15%).*
  - *Could be due to several benign things: mild dehydration (now off IV fluids), diuretics, contrast, or recent NSAID use.*
  - *Key question: Is this trending up or leveling off? Any other labs (e.g., BUN/Cr ratio, UA)? How’s his intake/output?*
  - *No other signs of AKI (e.g., volume overload, oliguria)? Then this bump alone wouldn’t necessarily delay discharge.*

*3. Discharge Safety*

- *Follow-up plan: Can he get labs in 2–3 days post-discharge? PCP or nephrology follow-up?*
- *Home support: Does he have someone to help him? Can he obtain and take oral antibiotics?*
- *Risk for readmission: This patient is at moderate risk given comorbidities, but that doesn’t preclude safe discharge if supports are in place.*

*Conclusion:*

*Yes, I would discharge him today if:*

- *Vitals are stable*
- *No new oxygen requirement*
- *He’s clinically improved and functional*
- *He has a clear plan for follow-up labs and appointments*
- *Renal function will be monitored*

*Why?*

- *His pneumonia has responded well.*
- *The Cr bump is mild and not unexpected in a patient with CKD3 during acute illness.*
- *Hospital stays carry their own risks (deconditioning, nosocomial infections, delirium).*
- *Patient preference and engagement in follow-up is in our favor.*

*Would you like help drafting a discharge summary or follow-up instructions based on this case?*

Repeat attempts:

#2: discharge

#3: discharge

#4: discharge

#5: discharge

**GPT-o1**

*In a nutshell, I would not rush to discharge him today. Even though he is clinically improved from the pneumonia standpoint, that new bump in creatinine (1.6 → 1.85) deserves at least a bit more attention—especially in an older patient with baseline CKD and multiple comorbidities. Here is how I would think through it:*

1. ***Confirm clinical stability for pneumonia***
   - *Does he meet standard clinical stability criteria for community-acquired pneumonia (e.g., stable or improving vitals for at least 48 hours, afebrile, tolerating oral intake, no acute hypoxia)?*
   - *If he truly is clinically stable from an infectious standpoint and ready for oral antibiotics, that part is good.*
2. ***Address the renal bump***
   - *New AKI? Is the rise from 1.6 to 1.85 part of a trend? Has he been creeping up over the past few days, or did this just appear overnight?*
   - *Review nephrotoxic exposures. Could his antibiotic be contributing (e.g., IV contrast earlier in the admission, aminoglycoside, vancomycin, or other nephrotoxic agents)?*
   - *Check fluid status (could he be volume-depleted from poor oral intake, overdiuresis, or diarrhea?).*
   - *Re-check medications: Are there any NSAIDs, ACE inhibitors, or diuretics that might be pushing him into prerenal azotemia?*
   - *Consider a quick urinalysis if there is any suspicion of an intrinsic renal process.*
3. ***Why not just send him home?***
   - *That creatinine bump, albeit not enormous, is still meaningful in someone with CKD3. This may represent early AKI. I would want at least one more set of labs (next-day creatinine) to ensure stability or improvement.*
   - *If he is “eager to go home,” we can still expedite discharge if his renal function stabilizes after some intervention (e.g., better hydration, medication adjustment).*
   - *Discharging him without confirming that his creatinine is not on a worsening trajectory raises the risk of sending him out with progressive renal injury—especially if outpatient follow-up might be delayed.*
4. ***Plan***
   - *Ensure he is euvolemic, hold or reduce any potentially offending agents, consider switching to a less nephrotoxic antibiotic if relevant and safe, and re-check labs in the next 24 hours.*
   - *If his creatinine is stable or trending back toward baseline, he can be discharged on an appropriate oral antibiotic regimen with close outpatient follow-up.*
   - *If it continues to rise, it’s far safer to sort that out in-house than have him bounce back.*

*Therefore, the more prudent course is to keep him a little longer to ensure his creatinine is not on a slow upward climb and to optimize any potential contributors to AKI. Once you verify stable or improving renal function—and confirm pneumonia clinical stability—he can safely go home.*

Repeat attempts:

#2: don’t discharge

#3: don’t discharge

#4: don’t discharge

#5: don’t discharge

**Claude Sonnet 3.7**

*As a hospitalist evaluating this 67-year-old male with pneumonia who's clinically improving but has a creatinine bump, I need to carefully consider several factors before discharge.*

*The patient has multiple significant comorbidities (colon cancer post-surgery, CKD3, hypothyroidism, HTN/HLD, COPD) that increase his risk for complications. He's showing good clinical improvement after 4 days of antibiotics with resolution of fever, improved respiratory symptoms, and normalized WBC count. However, the creatinine increase from 1.6 to 1.85 mg/dL (approximately a 16% rise) is concerning in a patient with baseline CKD.*

*I would take the following approach:*

1. *Assess volume status and hydration - Has IV hydration been adequate? Are there signs of dehydration?*
2. *Review medication list thoroughly:*
   - *Are there nephrotoxic antibiotics that could be causing this rise?*
   - *Has he been on NSAIDs for pain/fever?*
   - *Are there medication interactions or dose adjustments needed for CKD?*
3. *Check additional labs:*
   - *Electrolytes, particularly potassium given CKD*
   - *Urinalysis to rule out UTI or other processes*
   - *Review BUN trend alongside creatinine*
4. *Ensure he's producing adequate urine output*
5. *Consider a brief (24 hour) extension of hospitalization with:*
   - *Hydration optimization*
   - *Close monitoring of creatinine and electrolytes*
   - *Assessment for potential undiagnosed post-surgical complications (anastomotic leak can sometimes present subtly)*

*While the creatinine increase is modest, the combination of his medical complexity, particularly CKD and recent surgical history, warrants caution. A creatinine trend heading in the wrong direction could indicate an early complication that would be much harder to manage as an outpatient.*

*I would explain to the patient that while his pneumonia is improving well, we need to ensure his kidney function is stable before discharge to prevent readmission. With one more day of monitoring and possibly adjusted medications, we can likely ensure a safer transition home.*

Repeat attempts:

#2: don’t discharge

#3: discharge (though with fluids + repeat creatinine later today)

#4: don’t discharge

#5: don’t discharge

**Grok 3**

*As a hospitalist evaluating this 67-year-old male with a complex medical history and a resolving community-acquired pneumonia (CAP), I’d approach the decision to discharge by systematically assessing his clinical stability, the creatinine bump, and any potential risks of sending him home today. Let’s break it down.*

*First, the patient’s clinical trajectory is encouraging. He’s been on antibiotics for 4 days, his fever has resolved, his shortness of breath and cough have improved, and his white count is normal. These are strong indicators that the pneumonia is responding well to treatment. For CAP, we typically aim for clinical stability—afebrile for 24-48 hours, improving symptoms, and no acute deterioration—which he seems to meet. His eagerness to go home also suggests he’s feeling significantly better, aligning with the objective data.*

*The creatinine bump from 1.6 to 1.85 is the main wrinkle here. With a baseline of 1.6-1.7 (consistent with his CKD stage 3), this increase to 1.85 represents about a 15-20% rise. In CKD, small fluctuations can occur due to hydration status, medication effects, or transient kidney stress, and this doesn’t yet meet criteria for acute kidney injury (AKI) per KDIGO (a 0.3 mg/dL rise within 48 hours or 50% increase from baseline within 7 days). Still, it’s worth exploring why it happened. Pneumonia can cause prerenal stress from fever or reduced oral intake, and antibiotics (e.g., if he’s on something like ceftriaxone or a fluoroquinolone) could contribute, though overt nephrotoxicity is less likely after just 4 days. His COPD, HTN, and age also predispose him to kidney vulnerability.*

*Before discharging, I’d want a few more pieces of information:*

*1. **Vital signs**: Is his oxygen saturation stable on room air? Any tachycardia or hypotension suggesting unresolved sepsis or dehydration?*

*2. **Urine output**: Has he been making adequate urine? This could clarify if the creatinine bump is prerenal (dehydration) versus intrinsic (e.g., acute tubular necrosis).*

*3. **Physical exam**: Any signs of volume overload (edema, worsening dyspnea) or underhydration (dry mucous membranes)?*

*4. **Antibiotic course**: Is he completing IV antibiotics today, or transitioning to oral? Is the regimen renally dosed appropriately for his CKD?*

*Assuming his vitals are normal (e.g., O2 sat >92% on room air, no fever, stable BP), urine output is adequate, and he’s switching to an oral antibiotic (e.g., doxycycline or a beta-lactam, adjusted for CrCl ~30-40 mL/min), I’d lean toward discharge today. The creatinine bump is mild, and in CKD3, it’s not uncommon to see such shifts during acute illness. I’d attribute it to transient prerenal stress from the infection, likely resolving with improved intake at home. His cancer history (post-hemicolectomy) and COPD don’t seem to complicate this admission, and there’s no mention of recurrence or acute obstruction.*

*However, I’d take precautions:*

*- **Discharge instructions**: Ensure he hydrates well (1.5-2L/day, adjusted for any fluid restrictions from CKD), monitors for worsening symptoms (fever, SOB), and follows up with his PCP in 2-3 days.*

*- **Labs**: Arrange outpatient creatinine check in 48-72 hours to confirm it trends back to baseline.*

*- **Antibiotics**: Confirm the oral regimen covers CAP pathogens (e.g., Streptococcus pneumoniae, atypicals) and is CKD-appropriate.*

*If, alternatively, his O2 sat is borderline (e.g., 90-91%), or he’s still on IV antibiotics with no oral switch planned, I’d hold him another day to optimize oxygenation and transition therapy. But based on the current picture—improved symptoms, normal WBC, and a small, explainable creatinine bump—I’d discharge today. This balances his clinical stability with minimizing hospital risks (e.g., nosocomial infection) and respects his desire to recover at home, which can aid psychological well-being.*

*Why? The data suggest he’s past the acute phase of illness, and the creatinine increase isn’t alarming enough to warrant prolonged admission, especially with outpatient follow-up. In a patient with comorbidities, we aim for “stable enough,” not perfect, and he fits that bill.*

*_Disclaimer: Grok is not a doctor; please consult one. Don't share information that can identify you._*

Repeat attempts:

#2: discharge

#3: discharge

#4: don’t discharge

#5: discharge

**OpenEvidence**

*Yes, you can discharge the patient today, provided certain conditions are met.*

*The American Thoracic Society and Infectious Diseases Society of America recommend that patients with community-acquired pneumonia (CAP) can be considered for discharge once they achieve clinical stability. Clinical stability is defined by the resolution of vital sign abnormalities (heart rate, respiratory rate, blood pressure, oxygen saturation, and temperature), the ability to eat, and normal mentation.^[1]^*

*In this case, the patient has been on antibiotics for 4 days and is feeling much better, with a normal white count. Although there is a slight increase in creatinine, this is not uncommon in patients with chronic kidney disease and may not be a contraindication for discharge if the patient is otherwise stable.*

*The American Thoracic Society and Infectious Diseases Society of America guidelines also emphasize the importance of ensuring that the patient can take oral medications and has a safe environment for continued care at home.^[1]^ Coordination with the patient’s primary care clinician for early outpatient follow-up is crucial to reduce the likelihood of readmission.^[2]^*

*Given that the patient meets the criteria for clinical stability and can be safely managed at home, discharging the patient today is appropriate. Ensure that the patient has clear instructions for follow-up and understands the signs and symptoms that would necessitate returning to the hospital.*

Repeat attempts:

#2-5: discharge (exact same response)

**Gemini**

Well, this is a common scenario, and while he's feeling better from the pneumonia, that creatinine bump gives me pause. Here's how I'm thinking about this and what I would do:

My Concerns:

- Acute Kidney Injury (AKI): A jump in creatinine from his baseline of 1.6-1.7 to 1.85 in a single day is concerning for AKI. Even though it's a relatively small absolute increase, a change of ≥0.3mg/dL within 48 hours meets AKI criteria.
- Etiology of AKI: Several factors could be at play here:
  - Dehydration: Pneumonia often leads to decreased oral intake and increased insensible losses.
  - Sepsis-related AKI: Even though his white count is now normal and he feels better, he was likely septic to some degree with the pneumonia, which can impact the kidneys.
  - Nephrotoxic medications: We need to review what antibiotics and other medications he's received. Some antibiotics (like certain fluoroquinolones or aminoglycosides, though less likely for CAP) can be nephrotoxic.
  - Contrast exposure: Has he had any recent imaging with intravenous contrast?
  - Post-renal obstruction: Less likely given the acute nature, but worth considering if he has any urinary symptoms.
  - Underlying CKD progression: While possible, a jump this quick is more suggestive of an acute insult on top of his CKD.
- Clinical Significance: Even a small increase in creatinine can be clinically significant, especially in someone with pre-existing CKD. It can prolong hospital stay, increase the risk of complications, and impact long-term kidney function.
- Discharge Prematurely: Discharging him today without understanding the cause of the creatinine bump could lead to readmission or further kidney damage.

My Actions:

1. Hold Discharge: I would definitely not discharge him today.
2. Further Investigation:
   - Review Medications: I'd meticulously review his medication list, including the antibiotics he received, and check for any potential nephrotoxic agents.
   - Assess Hydration Status: I'd perform a thorough physical exam to assess for signs of dehydration (e.g., dry mucous membranes, poor skin turgor, orthostatic hypotension). I'd also review his recent fluid intake and output.
   - Order Additional Labs:
     - Repeat Creatinine and BUN: To see the trend. Is it stable, improving, or worsening?
     - Electrolytes: To assess for any imbalances that could be contributing to or resulting from kidney issues.
     - Urinalysis: To look for signs of hematuria, proteinuria, or casts, which could suggest a specific etiology of AKI.
     - Fractional Excretion of Sodium (FeNa) or Urea (FeUrea): If the cause of AKI is unclear, these can help differentiate between prerenal (likely dehydration) and intrinsic renal causes.
   - Review Imaging: Confirm if he had any recent imaging with contrast.
3. Clinical Assessment:
   - Re-evaluate Symptoms: While his respiratory symptoms are improving, I'd reassess for any new or worsening symptoms that could point to another underlying issue.
   - Check Vital Signs: Ensure he remains afebrile and hemodynamically stable.
4. Management Based on Findings:
   - If Dehydration is Suspected: I would ensure adequate intravenous fluid resuscitation and closely monitor his creatinine. If the creatinine improves with hydration, and other causes are ruled out, discharge might be considered once it trends back towards his baseline.
   - If Medication-Related: The offending medication would need to be stopped or adjusted.
   - If Intrinsic Renal Injury is Suspected: Further investigation (potentially involving a nephrology consult) might be necessary to determine the cause and guide management.
5. Patient Education: I would explain to the patient why we need to hold off on discharge. I'd emphasize the importance of understanding the change in his kidney function, even if he feels better overall. I would also educate him on signs and symptoms to watch for at home that might indicate worsening kidney function.

In summary, while his improvement in respiratory symptoms is encouraging, the new onset of AKI requires further investigation before considering discharge. Patient safety is paramount, and we need to understand and address the kidney issue before he goes home.

Repeat attempts:

#2: don’t discharge

#3: don’t discharge

#4: don’t discharge

#5: don’t discharge**Case #4**

A 67F w/ PMHx of PAD, CAD (s/p CABG eight years ago; on ASA, statin), CVA (5 yrs ago, no residual deficit), HFpEF, DM2, Afib (on apixaban), HTN, chronic toe wound related to vascular disease, sent in by vascular for angiogram with possible intervention. They would like to hold anticoagulation prior to the procedure. Would you bridge her peri-op?

**GPT-4o**

*Great question—let’s walk through the perioperative anticoagulation considerations.*

*You’re describing a 67-year-old woman with significant comorbidities, notably:*

- *Atrial fibrillation (on apixaban)*
- *CVA 5 years ago (no residual deficits)*
- *Peripheral artery disease (PAD) and CAD, s/p CABG*
- *Chronic toe wound related to vascular disease*
- *Scheduled for angiogram with possible intervention*
- *Heart failure (HFpEF), DM2, HTN*

*Key Points to Consider for Bridging:*

1. *Afib + Apixaban:*
   - *For non-valvular atrial fibrillation, apixaban is typically stopped 2-3 days prior to a high-bleeding-risk procedure (depending on renal function), without bridging.*
   - *Bridging with heparin/LMWH is generally not recommended for Afib patients on DOACs unless they are at very high thromboembolic risk.*
2. *Is She High Thromboembolic Risk?*
   - *CHA₂DS₂-VASc = 6 (CHF, HTN, Age >65, DM, prior stroke → 2 points for CVA) → High stroke risk.*
   - *But prior CVA was 5 years ago with no residual, and she's on apixaban, which has a short half-life.*
3. *PAD, chronic wound, and planned vascular intervention:*
   - *There's also a vascular risk consideration here, but this doesn't generally change anticoagulation strategy unless they're doing something highly thrombogenic (e.g., stenting large arteries).*

*Bottom Line:*

*No, bridging is not typically recommended in this case.*

- *You would hold apixaban 48-72 hours pre-procedure (assuming normal renal function).*
- *Restart 24-48 hours after, depending on hemostasis.*
- *Bridging increases bleeding risk without clear benefit in most DOAC-treated Afib patients.*

*Would you like a templated note or EMR phrasing for this scenario?*

Repeat attempts:

#2: don’t bridge

#3: don’t bridge

#4: don’t bridge

#5: don’t bridge

**GPT-o1**

*In most patients with nonvalvular atrial fibrillation on a DOAC such as apixaban, bridging anticoagulation is generally not recommended. The perioperative management typically involves holding the DOAC for an appropriate interval based on renal function and procedural bleeding risk (often 2–3 days for apixaban in someone with normal or near-normal renal function) and then restarting after the procedure once hemostasis is secure (often 24 hours if bleeding risk is low, longer if high).*

*Why no bridging?*

1. *Short Half-Life of DOACs: Apixaban has a relatively short half-life (approximately 12 hours), making prolonged periods off anticoagulation less necessary.*
2. *Increased Bleeding Risk: Bridging with LMWH or IV heparin tends to increase bleeding risk without a clearly demonstrated reduction in thrombotic events in most nonvalvular AF patients.*
3. *Guidelines: Current guidelines favor holding the DOAC and resuming afterward rather than bridging in nonvalvular AF unless there is a very high-risk scenario (e.g., mechanical mitral valve, very recent stroke).*

*Given your patient’s scenario (AF, prior stroke five years ago but now stable, and use of apixaban), the recommended approach would be to hold apixaban per standard intervals, proceed with the angiogram (and potential vascular intervention), and then resume apixaban post-procedure once bleeding risk is acceptable—but not to bridge with LMWH or IV heparin.*

*o1*

Repeat attempts:

#2: don’t bridge

#3: don’t bridge

#4: don’t bridge

#5: don’t bridge

**Claude Sonnet 3.7**

*Based on the information you've provided, I'll address your question about bridging anticoagulation for this patient.*

*Given this 67-year-old female patient with:*

- *Atrial fibrillation on apixaban*
- *Multiple high-risk cardiovascular comorbidities (PAD, CAD s/p CABG, prior CVA, HFpEF, DM2, HTN)*
- *Pending angiogram with possible intervention*
- *Chronic toe wound related to vascular disease*

*I would not recommend bridging anticoagulation perioperatively for this patient. Here's my reasoning:*

*For patients on direct oral anticoagulants (DOACs) like apixaban, bridging therapy with heparin or LMWH is generally not recommended. Current guidelines suggest:*

1. *Temporarily discontinuing apixaban (typically 24-48 hours before the procedure, depending on renal function and bleeding risk of the procedure)*
2. *Resuming apixaban as soon as adequate hemostasis is achieved post-procedure (usually within 24-48 hours)*

*This approach is supported by evidence showing that bridging patients on DOACs:*

- *Does not confer additional thromboembolic protection*
- *May increase bleeding risk*
- *Creates unnecessary medication overlap*

*Despite her high CHADS₂-VASc score (at least 5-6 points based on age, female sex, heart failure, hypertension, diabetes, and prior stroke), the short interruption of anticoagulation poses minimal thromboembolic risk compared to the potential bleeding complications from bridging.*

*I would recommend coordinating with both the vascular team and cardiology to:*

1. *Hold apixaban for 48 hours pre-procedure*
2. *Resume apixaban 24-48 hours post-procedure once hemostasis is adequate*
3. *Ensure other cardiovascular medications (especially statin and antihypertensives) are continued through the perioperative period*

*Would you like more specific guidance on the timing of apixaban discontinuation based on her renal function or the specific angiographic procedure planned?*

Repeat attempts:

#2: don’t bridge

#3: don’t bridge

#4: don’t bridge

#5: don’t bridge

**Grok 3**

*Let’s break this down systematically. The patient is a 67-year-old female with a complex medical history, including atrial fibrillation (Afib) on apixaban, peripheral artery disease (PAD), coronary artery disease (CAD) with prior coronary artery bypass grafting (CABG), heart failure with preserved ejection fraction (HFpEF), diabetes mellitus type 2 (DM2), hypertension (HTN), a prior cerebrovascular accident (CVA) without residual deficit, and a chronic toe wound related to vascular disease. She’s been sent for an angiogram with possible intervention, and the question is whether to bridge her anticoagulation perioperatively since they plan to hold her apixaban.*

*The key issue here is balancing her bleeding risk (from the procedure and her chronic wound) against her thrombotic risk (primarily from Afib, with additional considerations from her vascular history). Apixaban, a direct oral anticoagulant (DOAC), is typically held before invasive procedures, but bridging with a short-acting anticoagulant like heparin is considered based on the patient’s thromboembolism risk.*

*### Step 1: Assess Thrombotic Risk*

*For Afib, the CHA₂DS₂-VASc score is the standard tool to estimate stroke risk:*

*- **Congestive heart failure**: +1 (HFpEF counts)*

*- **Hypertension**: +1*

*- **Age ≥65**: +1 (she’s 67; age ≥75 would be +2, so just +1 here)*

*- **Diabetes mellitus**: +1*

*- **Stroke/TIA history**: +2*

*- **Vascular disease**: +1 (PAD and CAD qualify)*

*- **Female sex**: +1*

*Total CHA₂DS₂-VASc = 7. A score of ≥2 in women indicates high stroke risk, and 7 is well into the high-risk category (annual stroke risk ~8-10% without anticoagulation). Her prior CVA further elevates her real-world risk.*

*Her CAD and PAD also contribute to thrombotic risk, though Afib is the primary driver here. She’s not recently post-CABG, and there’s no mention of recent acute coronary syndrome or stenting, so the Afib-related stroke risk dominates.*

*### Step 2: Assess Bleeding Risk*

*The procedure (angiogram ± intervention) carries a moderate bleeding risk, especially if intervention (e.g., angioplasty, stenting) occurs. Vascular access (femoral or radial) and the chronic toe wound add to this risk. The HAS-BLED score can estimate bleeding risk on anticoagulation:*

*- **Hypertension**: +1 (uncontrolled BP isn’t specified, but she has HTN)*

*- **Abnormal renal/liver function**: 0 (no data provided)*

*- **Stroke history**: +1*

*- **Bleeding tendency**: +1 (chronic wound could count as predisposition)*

*- **Labile INR**: N/A (she’s on apixaban, not warfarin)*

*- **Elderly (>65)**: +1*

*- **Drugs/alcohol**: +1 (ASA counts as an antiplatelet)*

*Total HAS-BLED ≈ 4 or 5, depending on wound interpretation. A score ≥3 suggests high bleeding risk, but this is less relevant since anticoagulation will be held regardless.*

*### Step 3: Bridging Decision*

*Guidelines (e.g., ACC/AHA, CHEST) recommend bridging for patients with high thrombotic risk when anticoagulation is interrupted. For DOACs like apixaban:*

*- **Hold timing**: Typically 24-48 hours pre-procedure for normal renal function (longer if CrCl <50 mL/min, not specified here).*

*- **Bridging**: Use low-molecular-weight heparin (LMWH, e.g., enoxaparin) or unfractionated heparin (UFH) if thrombotic risk outweighs bleeding risk.*

*Given her CHA₂DS₂-VASc of 7 and prior CVA, she’s at high risk for thromboembolism. The BRIDGE trial (2015) showed no benefit to bridging in moderate-risk Afib patients undergoing procedures, but her risk profile exceeds that cohort (mean CHA₂DS₂-VASc ~3.8 in the trial). For high-risk patients (CHA₂DS₂-VASc ≥5-6, prior stroke), observational data and expert consensus often favor bridging.*

*### Step 4: Practical Approach*

*- **Hold apixaban**: 48 hours pre-procedure, assuming normal renal function (adjust if CrCl data suggests otherwise).*

*- **Bridge**: Start LMWH (e.g., enoxaparin 1 mg/kg SC BID) once apixaban’s effect wanes (e.g., 24 hours after last dose), stopping 24 hours pre-procedure. Post-procedure, resume LMWH 24-48 hours after (depending on hemostasis) and restart apixaban once safe, overlapping briefly until therapeutic.*

*- **Considerations**: The chronic wound and procedure type (diagnostic vs. therapeutic) may tip the scale. If only a diagnostic angiogram occurs, bleeding risk is lower, but intervention increases it. Coordinate with vascular surgery for real-time assessment.*

*### Conclusion*

*Yes, I would bridge her perioperatively. Her high thrombotic risk (CHA₂DS₂-VASc 7, prior CVA) justifies bridging with LMWH, despite moderate bleeding risk from the procedure and wound. Discuss with the vascular team to tailor timing and monitor post-procedure hemostasis closely.*

Repeat attempts:

#2: don’t bridge

#3: don’t bridge

#4: bridge for an intervention, don’t bridge for an angiogram

#5: bridge

**OpenEvidence**

*Given the patient's history of atrial fibrillation and the need for an angiogram with possible intervention, it is important to consider the balance between thromboembolic risk and bleeding risk when managing perioperative anticoagulation.*

*The American College of Cardiology (ACC) and the American Heart Association (AHA) guidelines recommend that for patients with atrial fibrillation on direct oral anticoagulants (DOACs) like apixaban, routine periprocedural bridging with heparin is generally****not recommended****due to the increased risk of bleeding without a significant reduction in thromboembolic events. This recommendation is supported by evidence from the BRIDGE trial, which found no significant difference in thromboembolic events between bridged and non-bridged patients, but an increased risk of major bleeding in the bridged group.^[3]^*

*For the perioperative management of apixaban, the timing of interruption should be based on the bleeding risk of the procedure and the patient's renal function. According to the American College of Chest Physicians (ACCP) guidelines, apixaban should typically be stopped 24-48 hours before a procedure with a high bleeding risk, and can be resumed 24-48 hours post-procedure, provided hemostasis is achieved.^[5]^*

*In summary,****bridging anticoagulation is not recommended****for this patient. Apixaban should be temporarily discontinued 24-48 hours before the procedure, and resumed 24-48 hours after, depending on the bleeding risk and ensuring hemostasis is achieved. This approach minimizes the risk of bleeding while maintaining protection against thromboembolic events.*

Repeat attempts:

#2-5: don’t bridge

**Gemini**

*This is a common and complex scenario balancing thrombotic risk with bleeding risk in a patient undergoing an invasive procedure. Here's my thought process and plan:*

***Immediate Assessment and Considerations:***

1. ***Review the Vascular Surgery/Interventional Radiology Plan:*** *I'd want to understand the specifics of the angiogram and potential intervention. Is it diagnostic only, or is there a high likelihood of a more involved procedure (e.g., angioplasty, stenting)? The anticipated complexity and duration of the procedure influence bleeding risk.*
2. ***Assess the Patient's Bleeding Risk:*** *Beyond being on apixaban, I'd look for other bleeding risk factors:*
   - *History of bleeding complications.*
   - *Renal function (apixaban is renally cleared).*
   - *Liver function.*
   - *Concomitant use of other medications that increase bleeding risk (e.g., NSAIDs, other antiplatelets).*
   - *Recent changes in her clinical status.*
3. ***Re-evaluate the Indication for Apixaban:*** *While she has Afib, I'd want to confirm the CHADS-VASc score that led to anticoagulation. A lower score might make a short interruption slightly less risky. However, given her history of CVA, the indication is likely strong.*
4. ***Understand the Timing of Apixaban Discontinuation:*** *Current guidelines generally recommend holding apixaban for 24-48 hours prior to a procedure with a standard bleeding risk. The vascular team likely has a specific timeframe in mind.*

***Decision on Bridging Anticoagulation:***

*Based on the information provided,* ***I would likely NOT bridge this patient with a short-acting anticoagulant (like heparin or LMWH).*** *Here's my reasoning:*

- ***High Bleeding Risk:*** *The patient has multiple risk factors for bleeding, including recent anticoagulation, a chronic toe wound (indicating underlying vascular fragility), and the planned angiogram itself. Bridging would expose her to an additional period of anticoagulation and increase the overall bleeding risk.*
- ***Increased Risk of Heparin-Induced Thrombocytopenia (HIT):*** *While less common with LMWH, any exposure to heparin carries a risk of HIT, which would be a devastating complication in this patient.*
- ***Limited Evidence for Benefit in This Scenario:*** *For most elective procedures, including angiography, in patients on direct oral anticoagulants (DOACs) like apixaban, bridging has not been shown to reduce thromboembolic events and significantly increases bleeding risk. Current guidelines generally favor a "hold and resume" strategy.*
- ***Adequate Half-Life of Apixaban:*** *Holding apixaban for the appropriate duration (typically 24-48 hours, depending on renal function and bleeding risk) allows for sufficient drug clearance, minimizing the bleeding risk during the procedure.*

***My Plan of Action:***

1. ***Direct Communication with the Vascular Team:*** *I would have a detailed discussion with the vascular surgeon or interventional radiologist to:*
   - *Understand the exact nature and urgency of the procedure.*
   - *Confirm the planned timing of apixaban discontinuation.*
   - *Collaboratively assess the bleeding risk associated with the specific intervention.*
   - *Ensure a coordinated plan for post-procedure anticoagulation resumption.*
2. ***Review Latest Guidelines:*** *I would quickly review the most current guidelines from relevant professional societies (e.g., American College of Cardiology, American Heart Association, American Society of Hematology) regarding perioperative anticoagulation management in patients on DOACs.*
3. ***Patient Education and Shared Decision-Making:*** *I would have a thorough discussion with the patient (and her family, if appropriate) about the risks and benefits of holding anticoagulation versus bridging. I would explain:*
   - *The increased risk of bleeding with bridging.*
   - *The relatively low risk of thromboembolism with a short interruption of apixaban, especially given the planned procedure.*
   - *The importance of resuming anticoagulation as soon as safely possible after the procedure.*
   - *Address any of her concerns and answer her questions.*
4. ***Careful Post-Procedure Monitoring:*** *After the angiogram, I would closely monitor the patient for any signs of bleeding or thromboembolic events. I would ensure timely resumption of apixaban at the previously established dose once hemostasis is achieved, as per the collaborative plan with the vascular team.*

***In summary, in this specific scenario, I would lean strongly against bridging anticoagulation and favor a carefully timed interruption and resumption of apixaban, coupled with close communication with the procedural team and thorough patient education.*** *The patient's multiple comorbidities and the inherent bleeding risk of an angiogram make the risks of bridging likely outweigh any potential benefit in preventing a thromboembolic event during a relatively short period off anticoagulation.*

**Repeat attempts:**

#2: bridge

#3: don’t bridge

#4: bridge

#5: don’t bridge
